# Broadband diffusion metasurface based on a single anisotropic element and optimized by the Simulated Annealing algorithm

**DOI:** 10.1038/srep23896

**Published:** 2016-04-01

**Authors:** Yi Zhao, Xiangyu Cao, Jun Gao, Yu Sun, Huanhuan Yang, Xiao Liu, Yulong Zhou, Tong Han, Wei Chen

**Affiliations:** 1 Information and Navigation Institute, Air Force Engineering University, Xi’an,710077, China; 2 Department of Electronic Engineering, Tsinghua University, Beijing, 10084, China

## Abstract

We propose a new strategy to design broadband and wide angle diffusion metasurfaces. An anisotropic structure which has opposite phases under x- and y-polarized incidence is employed as the “0” and “1” elements base on the concept of coding metamaterial. To obtain a uniform backward scattering under normal incidence, Simulated Annealing algorithm is utilized in this paper to calculate the optimal layout. The proposed method provides an efficient way to design diffusion metasurface with a simple structure, which has been proved by both simulations and measurements.

A metasurface is an ultrathin planar artificial structure which is composed of sub-wavelength particles. Recently, it has been of great interest both in academic and engineering field owning to its extraordinary capability of manipulating electromagnetic wave at will. By introducing abrupt phase shift to the surface, numerous functions can be achieved such as anomalous reflection/refraction^1^,^2^, polarization conversion^3^,^4^,^5^,^6^, lensing^7^, propagating wave to surface wave conversion^8^ and so on. One of the potential applications of metasurface is to reduce the radar cross section (RCS) of an object, which is of great significance in military field.

Generally, there are two different ways to achieve RCS reduction. One of them is the absorptive method that transforms the scattering energy into heat. The other is reshaping the scattering pattern to suppress the detected level within a critical area. For the absorptive method, the typical implement is called Salisbury screen, which employs a resistive sheet put above a metallic surface with a distance of a quarter of the wavelength^9^. However, its bulky structure and narrow working band hinder its practical application. High impedance surfaces loaded with lumped resistances^10^ and perfect absorbing metamaterial with lossy substrate^11^ offer an effective way to reduce the thickness of the structure, but the band is still limited since their working principle is based on structure resonance. For the reshaping scattering pattern method, the most direct way is to reshape the appearance of an object. But this approach is extremely restricted by the other engineering aspects such as weight, volume and aerodynamic performance. Consequently, it is necessary to develop thin planar metasurface to control the scattering pattern without changing the appearance of an object dramatically.

In 2007, Panquay *et al*. proposed a checkerboard-like structure using a staggered combination of artificial magnetic conductors (AMC) and perfect electric conductors (PEC)^12^. The surface successfully disperses the scattering energy into several off-normal directions. However, this design shows a narrow band RCS reduction just around the frequency of AMC resonance. To broaden the working band, a combination of dual-AMC structure working at different frequencies is introduced to compose the chessboard^13^,^14^,^15^,^16^. All the chessboard structures show a common scattering pattern: a low scattering area around normal direction with four lobes in the diagonal planes. For bistatic detection, the strong lobes can be easily detected. In order to suppress these lobes, a metasurface that can scatter the incoming energy in all directions is required. Recently, this goal has been achieved by the metasurface with randomly arranged layout^17^,^18^,^19^,^20^. In refs 17, 18, 19, diffusion metasurfaces are developed by using units of various dimensions. The phase of each unit is chosen to be distributed randomly, leading to low RCS in broadband and wide angle. Reference 20 demonstrates a diffusion metasurface by perturbing the periodic arrangement of the AMC tiles. It is shown that the aperiodic structure has 3-dB lower maximum bistatic RCS compared to the periodic one. More recently, the concept of coding metamaterial has been reported^21^,^22^. Scattering patterns can be manipulated by designing the “0” and “1” sequences of digital elements. Based on this idea, diffusion metasurfaces have been realized with the aid of optimization methods^18^,^23^,^24^.

At present, polarization conversion surface are widely studied. It is composed of an anisotropic structure which shows diverse resonant states according to incident polarization states^25^,^26^,^27^. Reference 28 proposed a transmissive metasurface composed by anisotropic nanoposts to achieve complete control of waves. Inspired by this property, we exploit a single anisotropic element to build broadband diffusion metasurface. Instead of employing several different isotropic elements, the necessary phase difference can be obtained by simply rotating the anisotropic structures. Moreover, In order to obtain the uniform backward scattering, Simulated Annealing algorithm is utilized in this paper to calculate the optimal layout. The proposed method provides an efficient way to design diffusion metasurfaces with simple structures, which is confirmed by both simulations and measurements.

## Results

### Anisotropic element, layout optimization and diffusion metasurface

**Figure 1:**
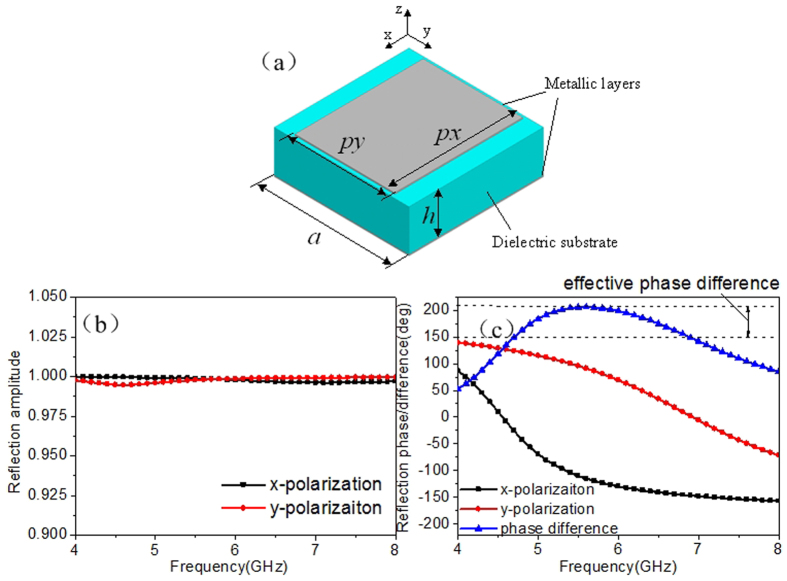
Geometry of anisotropic element and its reflection properties. (**a**) Geometry of anisotropic element. The dimensions of the structure are a = 10 mm, px = 9.5 mm, py = 7.4 mm and h = 3 mm. The reflection phases of x- and y-polarized incident waves can be easily adjusted by changing px and py, respectively. (**b**) Reflection amplitude versus frequency. (**c**) Reflection phases for both x- and y-polarizations and their difference.

To our knowledge, arbitrary asymmetric geometry leads to different phase responses under the x- and y-polarized incident wave. To concisely demonstrate our design method and facilitate the simulation procedure, we choose a simple rectangular patch structure as candidate, which is shown in Fig. 1a. The middle layer is a dielectric substrate with dielectric constant 2.65 and loss tangent 0.001. The bottom of the substrate is covered with a full metallic ground. Figure 1b,c depict the reflection characters of the structure calculated by Ansys HFSS. Due to full metallic ground on the back, the entire structure is perfectly reflective for incident waves. The amplitude remains above 0.99 for both x- and y-polarized incidence as shown in Fig. 1b. Figure 1c gives the reflection phases of both polarizations as well as their difference. One can see that the structure yields around 180° phase difference over a broad spectral band. Based on the concept of coding metamaterial, for normal incidence of given polarization, we can nominate the structure as “0” element while just rotate it by 90° along its center axis to obtain “1” element. To satisfy the periodic boundary in the element simulation, a lattice which contains 4 × 4 anisotropic elements in the same orientation is generated. Hence, the different coding sequence can be translated into orientation of lattices (90° rotation or not) for final fabrication.

Once the lattice has been prepared, we are going to seek for the optimal layout of the diffusion metasurface. It is known that a traditional conducting surface has a uniform reflection phase when a plane wave impinges on it, leading to a strong directive scattering pattern. In order to manipulate the reflect beam direction, a phase gradient is introduced into the interface to fully control the equiphase wavefront based on the generalized Snell’s law^1^. Here, our goal is to redirect the scattering energy in all directions to minimize directional reflection. Based on this idea, the reflection phase from each part of the surface should be distorted as much as possible rather than in an equal or gradient shifting manner. The simplest way is to generate a matrix of random phase distribution. However, it cannot guarantee an optimal result and the continuous changed phases are hard to yield in reality. Here, we adopt a comprehensive approach to solve this issue that includes array theory, coding matrix and optimization algorithm. Considering a M × N array composed of lattices of opposite reflection phase (0° or 180°) but equal magnitude under normal incidence. According to the array theory^29^, the total scattering field of the metasurface can be expressed as


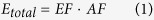


where *EF* represents the pattern function of a lattice. In our model, we assume that the *EF* is fixed. *AF* represents the array factor which can be described as


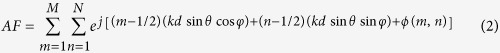


where *d* is the distance between two adjacent lattices, *θ* and *φ* are the elevation and azimuth angles, respectively. *ϕ*(*m, n*) is the reflection phase of each lattice which can be translated into “0” or “1”. Then, the whole surface can be represented by a M × N coding matrix. Here, we employ a Simulated Annealing algorithm to find the optimal coding matrix due to its merits of simple description and high efficiency.

**Figure 2:**
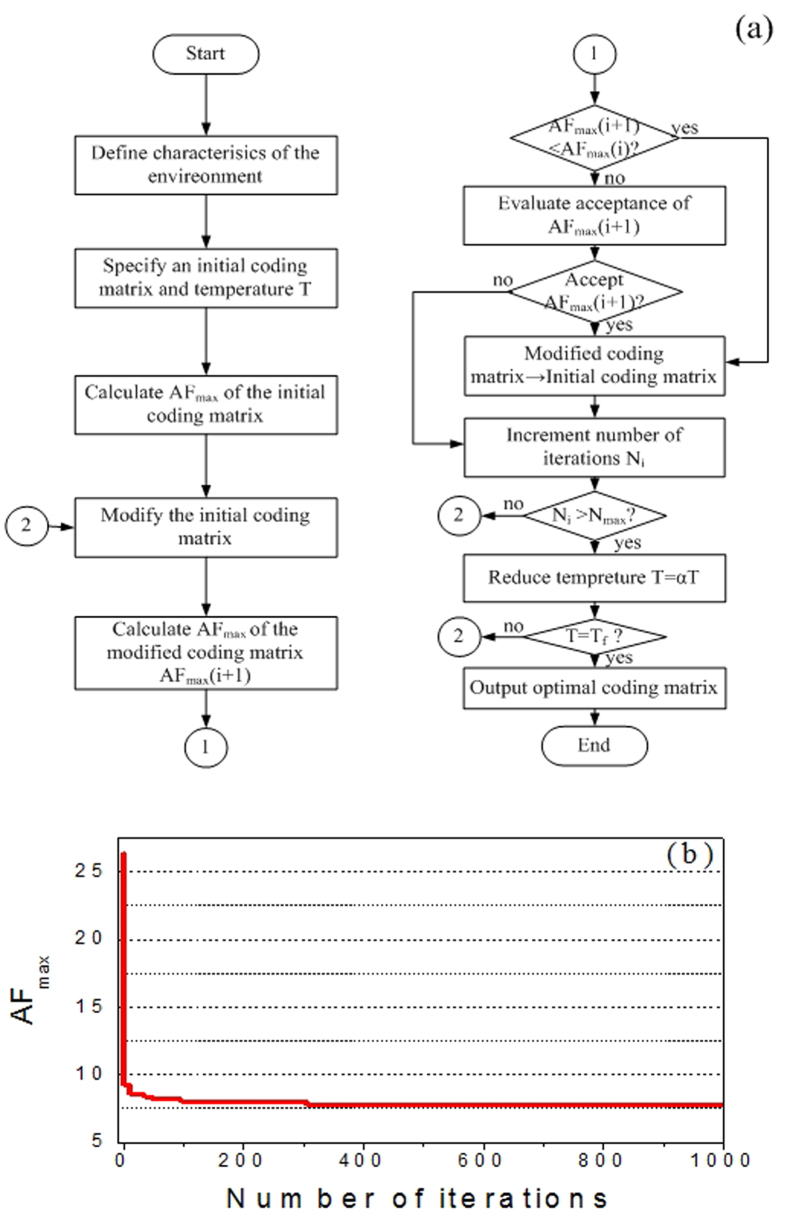
The Simulated Annealing algorithm for finding the optimal coding matrix. (**a**) Flowchart of the optimization algorithm. (**b**) The evolution plot of AF_max_.

**Figure 3:**
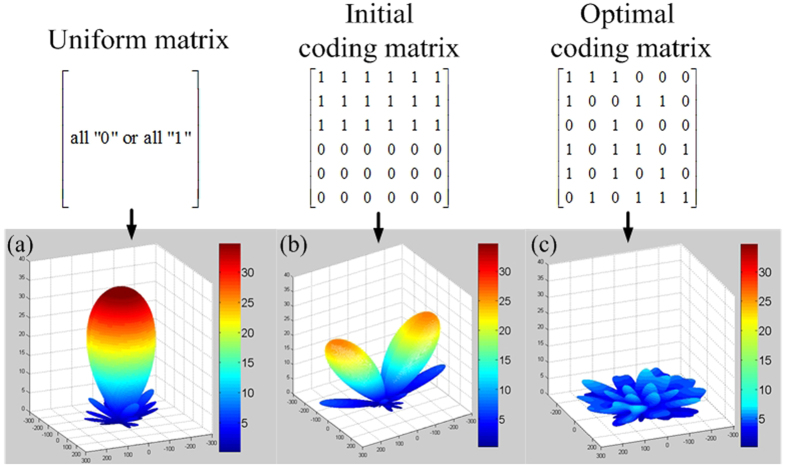
Coding matrices and their corresponding patterns. (**a**) Uniform matrix. The matrix is composed of all “0” or all “1” elements, resulting in strong reflected beam at normal direction. (**b**) Initial coding matrix. The matrix is composed of equal number of “0” and “1” which arranged in the lower half and upper half. Its pattern shows two symmetric beams at oblique directions. (**c**) Optimal coding matrix. The coding matrix is composed of “0” and “1” in optimized distribution. There is no obvious beam in its pattern.

Simulated Annealing is a method for local searching proposed by Kirkpatrick^30^. It begins with an initial solution that is randomly modified in an iterative process. The main parameters of Simulated Annealing are the initial temperature T, the decreasing rate in each iteration *α*, the final temperature T_f_, the number of iterations I and the merit function. In our model, we define an initial coding matrix with equal number of “0” and “1”. Then it is upgraded by exchanging the positions of an arbitrary pair of “0” and “1”. The parameters T, *α*, T_f_ and I are set as 100, 0.9, 0 and 1000, respectively. For low RCS performance, good diffusion pattern is expected. Thus, our goal is to find the optimal coding matrix(*M*_*best*_) that leading to a desired scattering field with smallest maximum value. Thus, the issue is a min-max problem in which the merit function can be expressed as F(*M*_*best*_) = min(*AF*_max_), where *AF*_*max*_ is the maximum value of *AF* corresponding to given coding matrix. Figure 2(a) shows the flow chart of the proposed method for optimizing the coding matrix. Generally, the bigger the array size, the better the obtained diffusion effect. However, considering the time cost of the simulation procedure and the overall scale of the metasurface, we deem that an array which contains 6 × 6 lattices (M = N = 6) is appropriate to illustrate our design method. The evolution plot of *AF*_*max*_ is given in Fig. 2(b). It can be seen that the curve declines rapidly during the inception phase and then it tends to stabilize, proving the high efficiency of the algorithm. Furthermore, compared with the *AF*_*max*_ of initial coding matrix, the final *AF*_*max*_ gets significant suppressed after optimization. As a result, the maximum value of the scattered field can be reduced due to energy conversation. Figure 3 show the calculated scattering fields of the uniform coding matrix (representing metallic surface), the initial coding matrix and the optimal coding matrix. It can be seen clearly that the uniform coding matrix leads to a strong reflection beam toward the normal direction, while the pattern of initial coding matrix presents two main beams as illustrated in Fig. 3a,b, respectively. The optimal coding matrix and its pattern are shown in Fig. 3c. It can be clearly seen that our model successfully finds an optimized solution which leads to a perfect diffusion pattern.

**Figure 4:**
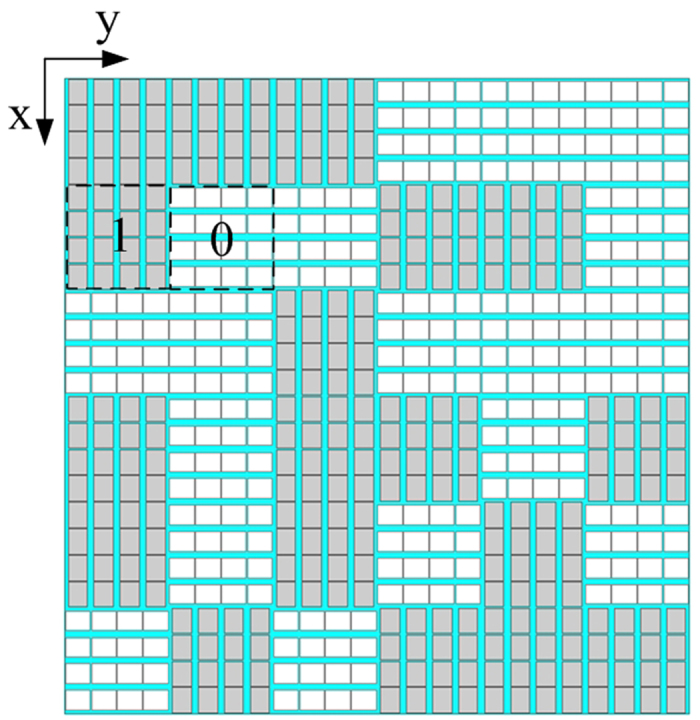
Geometry of proposed diffusion metasurface. For an intuitive view, the different oriented lattices are distinguished by gray and white colors.

By employing the proposed anisotropic element and the optimal coding matrix, the metasurface is built up as shown in Fig. 4. In our model, we choose the lattice with x-oriented patches as “1” element, while its orthogonally arranged counterpart is “0” element. Of cause, interchanging the assignment would also be acceptable. The overall dimensions of the metasurafce is 240 mm × 240 mm × 3 mm (about 4.6*λ* × 4.6*λ* × 0.058*λ, λ* is the wavelength of incident wave at 5.8 GHz).

### Simulations and measurement

**Figure 5:**
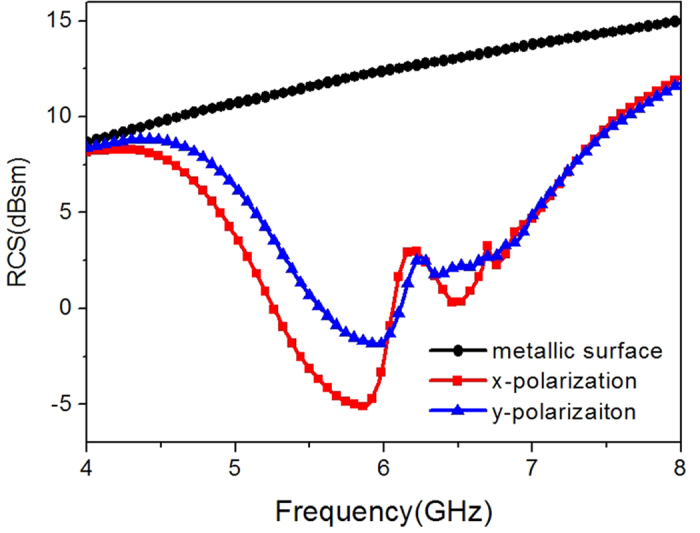
Simulated RCS for normal incidence of both x- and y-polarization.

**Figure 6:**
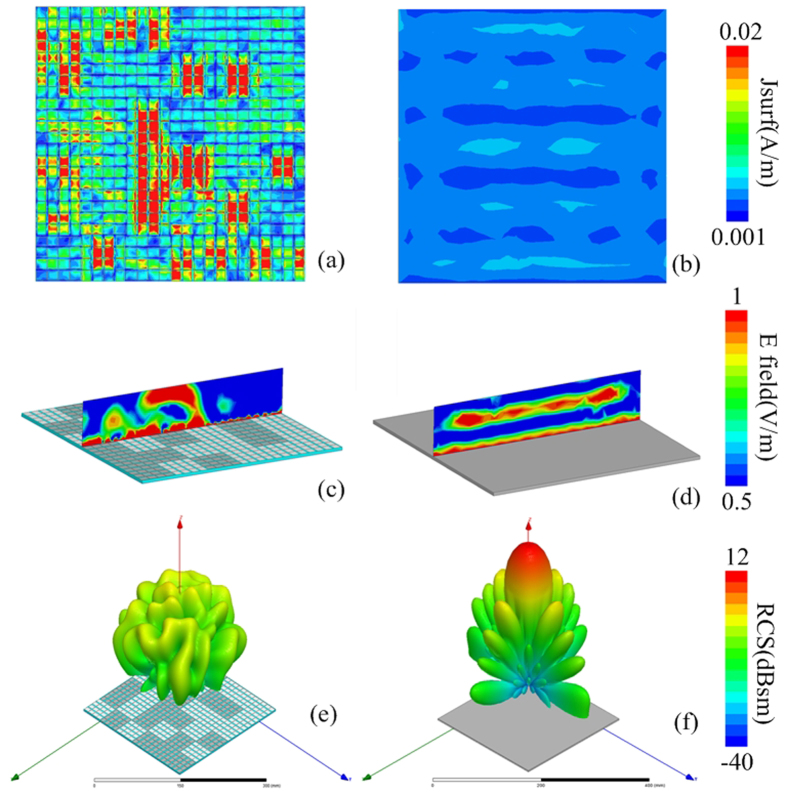
Simulated near-field and far-field patterns under x-polarized normal incidence. (**a,b**) The surface current distributions of the metasurface and metallic surface, respectively. (**c,d**) The near-electric-field distributions. (**e,f**) The far-field scattering patterns.

Figure 5 shows the RCS of the metasurface as well as a same-size metallic surface with plane wave normally impinging. Obvious reduction is obtained from 4–8 GHz for both polarizations. The 10 dB reduction band is 5.2 GHz–6.86 GHz and 5.44 GHz–6.9 GHz for x- and y-polarized incident wave, respectively. The most sufficient cancelation happens when the phase difference of the different lattices is exactly 180°, causing the two dips in the RCS curve. The frequency deviation between the dips and the 180° phase difference is attributed to the different boundary conditions. Figure 6a,b show the surface current distributions of proposed metasurface and the same-size metallic surface under normal incidence at 5.86 GHz. It demonstrates that the elements on the metasurface present different resonant states, which is critical to disturb the equiphase reflection (see Fig. 6c,d). As a result, the metasurface generates a diffuse scattering pattern in the far-field with suppressed amplitude as depicted in Fig. 6e,f. The patterns obtained by full wave simulation are in agreement with the results of the optimization algorithm, proving the effectiveness of our design method.

**Figure 7:**
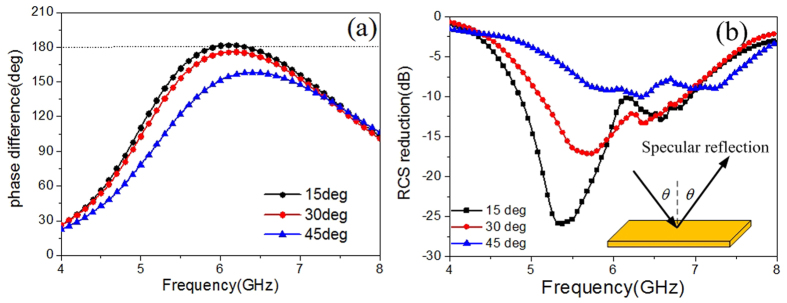
Angular performances of the metasurface. (**a**) The phase difference of the anisotropic element under oblique incidences. (**b**) The RCS reduction versus frequency. The observation angle is equal to the incident angle to detect the specular reflection.

**Figure 8:**
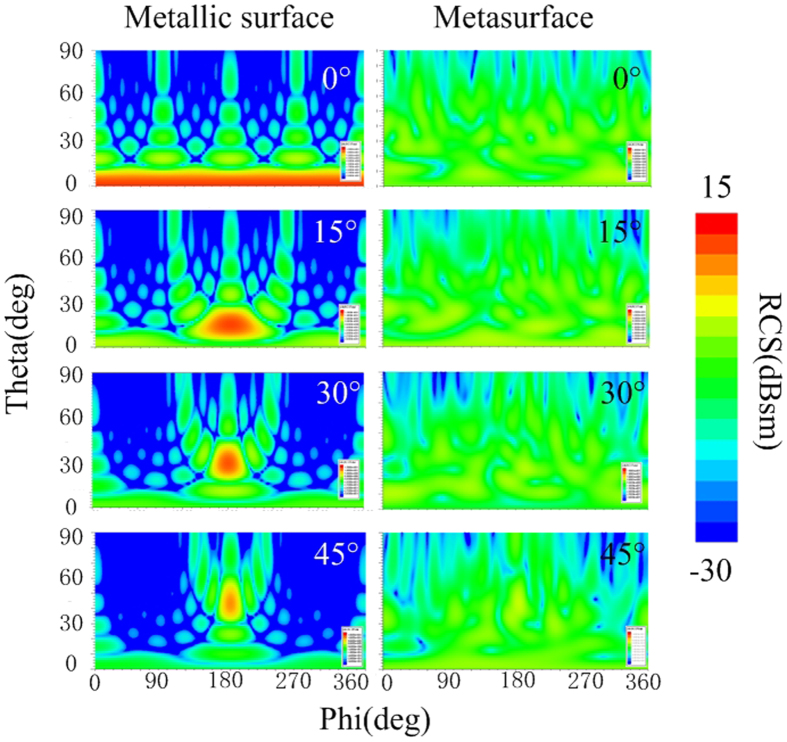
Scattering spectra in the backward space. The spectra are depicted in the same color map scale at 5.86 GHz. The observation area is 0°–90° in elevation and 0°–360° in azimuth.

Angular performances are investigated to give a comprehensive understanding of our metasurface. TM waves impinging from 15°, 30°, and 45° in the xoz plane are considered. Figure 7a shows that the phase difference decreases apparently along with the incident angle increases, proving the sensitivity of the anisotropic element to incident angles. Figure 7b shows the RCS reduction at the specular directions versus frequency. It can be observed that the specular reflection can be suppressed in broadband and wide angles. Along with the incident angle increases, the dips shift and the suppression effect is weakened, which is attributed to the phase difference alteration. To verify the diffusion effect of our metasurface, the scattering spectra in the backward space are shown in Fig. 8. The results of the metallic surface are also shown in the same color map scale for comparison. It reveals that the suppression of specular reflections is achieved in wide angles through distributing most of the scattered energy to various off-specular directions. It is worth noting that though the scattering may get enhanced at some directions, the values are small enough to avoid being detected, providing a viable solution in practice.

**Figure 9:**
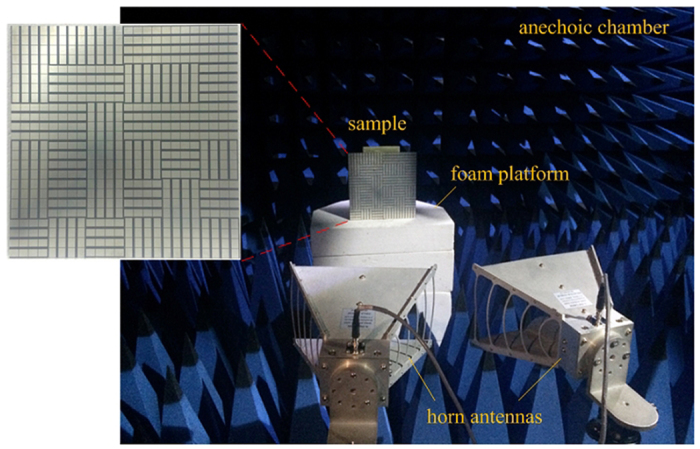
The fabricated metasurface and measurement setup. The height of the sample is kept the same with the antennas and the distance is far enough to satisfy the far field measurement requirement. For normal incidence, nearly 5 degree is maintained in practice due to the volume of the horn antennas.

**Figure 10:**
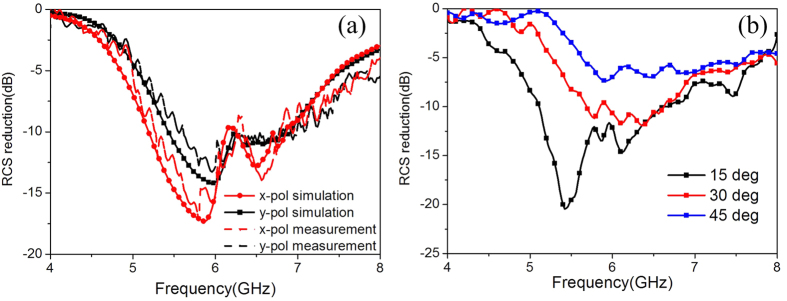
RCS reduction versus frequency. (**a**) The measurement results under normal incidence. The corresponding simulated results are also depicted for comparison. (**b**) The measurement results under oblique incidence. For all the measured results in (**a**,**b**), the reflectivity of the metasurface and the metallic board are separately measured first. Then, subtraction is made between their values.

To validate the predicted performance of our diffusion metasurface, a sample is fabricated and its performance under normal incidence is measured. Figure 9 shows the photo of the sample and the measurement setup. The sample is manufactured by printed circuit board processing technology. The dielectric substrate is F4B board with a dielectric constant 2.65 and a loss tangent 0.001. The metal patches and ground are 0.036 mm-thick copper layers. For measurement, two identical horn antennas are utilized as transmitting and receiving devices, respectively. Then, the scattering performance is evaluated by the transmission coefficients obtained by vector network analyzer. A same-size metallic board is also measured as reference. Figure 10(a) shows the measured RCS reduction versus frequency under normal incidence. The simulated results are also depicted. The measured 10 dB RCS reduction bandwidths for the x- and y-polarizations are 5.26–6.80 GHz and 5.56–6.82 GHz, respectively. The fractional bandwidth for 6 dB reduction is more than 35% for both cases. Good agreement can be found between the results of measurement and simulation. The results of oblique incidence are shown in Fig. 10(b). With the of the incident angle increase, the change tendency is consistent with the simulation results. The deviation in value can be attributed to the measurement and fabrication errors. At this point, the good performance of the proposed metasurface is confirmed.

## Conclusion

A thin diffusion metasurface has been proposed for RCS reduction in broadband and wide angle. An anisotropic element was employed in the metasurface based on its two-state reflection property, which can be assigned as “0” and “1” according the concept of coding metamaterial. Array theory and Simulated Annealing algorithm were adopted to optimize the coding matrix mathematically and find the best layout. A sample was built up for full-wave simulation and fabricated for measurement. Both the simulated and measured results demonstrated the effectiveness of the proposed metasurface. It is worth mentioning that we choose the rectangular patch structure as basic element due to its simple geometry. It can be modified or replaced by other anisotropic structures with better performances on band or angular stability, so that the performance of the entire metasurface can be enhanced further. On the other hand, in our work we choose a 6 × 6 array size considering the efficiency of optimization procedure and computing capability of our hardware. We believe that better performance can be achieved by expanding the array size.

## Methods

Backscattering measurement is conducted in an anechoic chamber to minimize interference from the environment. For normal incidence, two identical horn antennas are placed adjacently in front of the sample with a distance of 1.5 m to meet the condition of far-field test, so that the beam from the transmitting antenna can be approximated as plane wave. The transmitting and receiving antennas are connected to the two ports of Agilent N5230C VNA. Then, the reflection performance can be evaluated by S21 parameter. To further eliminate the noises in the environment, gate-reflect-line calibration in time-domain analysis kit of VNA is employed. We rotate the sample around its central axis to obtain its performances under different polarizations. For oblique incidence, the transmitting and receiving antennas are set as TM polarization and placed with a same angle with respect to the normal direction of the sample.

## Additional Information

**How to cite this article**: Zhao, Y. *et al*. Broadband diffusion metasurface based on a single anisotropic element and optimized by the Simulated Annealing algorithm. *Sci. Rep.* **6**, 23896; doi: 10.1038/srep23896 (2016).
